# Molecular Cloning and Characterization of Novel Phytocystatin Gene from Turmeric, *Curcuma longa*

**DOI:** 10.1155/2014/973790

**Published:** 2015-04-02

**Authors:** Seow-Neng Chan, Norliza Abu Bakar, Maziah Mahmood, Chai-Ling Ho, Noor Azmi Shaharuddin

**Affiliations:** ^1^Department of Biochemistry, Faculty of Biotechnology and Biomolecular Sciences, Universiti Putra Malaysia (UPM), 43400 Serdang, Malaysia; ^2^Biotechnology Research Center, Malaysian Agricultural Research and Development Institute (MARDI), 43400 Serdang, Malaysia; ^3^Department of Cell and Molecular Biology, Faculty of Biotechnology and Biomolecular Sciences, Universiti Putra Malaysia (UPM), 43400 Serdang, Malaysia

## Abstract

Phytocystatin, a type of protease inhibitor (PI), plays major roles in plant defense mechanisms and has been reported to show antipathogenic properties and plant stress tolerance. Recombinant plant PIs are gaining popularity as potential candidates in engineering of crop protection and in synthesizing medicine. It is therefore crucial to identify PI from novel sources like *Curcuma longa* as it is more effective in combating against pathogens due to its novelty. In this study, a novel cDNA fragment encoding phytocystatin was isolated using degenerate PCR primers, designed from consensus regions of phytocystatin from other plant species. A full-length cDNA of the phytocystatin gene, designated *CypCl*, was acquired using 5′/3′ rapid amplification of cDNA ends method and it has been deposited in NCBI database (accession number KF545954.1). It has a 687 bp long open reading frame (ORF) which encodes 228 amino acids. BLAST result indicated that *CypCl* is similar to cystatin protease inhibitor from *Cucumis sativus* with 74% max identity. Sequence analysis showed that *CypCl* contains most of the motifs found in a cystatin, including a G residue, LARFAV-, QxVxG sequence, PW dipeptide, and SNSL sequence at C-terminal extension. Phylogenetic studies also showed that *CypCl* is related to phytocystatin from *Elaeis guineensis*.

## 1. Introduction 

In an effort to increase food security and provide protection to plants that are constantly in combat with pest and pathogens, various genes coding for defense related proteins have been incorporated into crops. An example that gains popularity as a suitable candidate gene for crop protection is protease inhibitors (PIs). A great number of PIs have been characterized from different plant species and engineered into crop plants for protections against pest and pathogens [1–4]. PIs are commonly found in plants and play a role in regulating digestive proteases by forming a complex with the target protein, either on the active site or on the allosteric site [4]. In a nonhost plant pathogenesis, PIs are released as one of the defense responses by plants to protect themselves against the attacks of their predators [3–5]. However, as pest and pathogens are slowly adapting and acquiring resistance against the currently utilised PIs, it is crucial to continuously discover PIs from novel sources. PIs from novel sources have not been exposed to the crop pest and pathogens before and this helps in overcoming the resistance adaptation by pest and pathogens [3, 4].

Cysteine protease inhibitor or phytocystatin is a type of PI that has long been identified and reported to have important roles in plants [6]. It acts as an endogenous proteolysis regulator during seed maturation and development, involves in programmed cell death, and inhibits exogenous proteases released by plant predators like insects, nematode, bacteria, and pathogens during the attack [6–12]. In recent years, however, phytocystatin has also been suggested to be involved in response to biotic and abiotic stresses as they are highly expressed during harsh condition such as cold, drought, salt stress, oxidant stress, and wounds [8, 9, 12]. All these properties encourage more research to be done on PIs, especially on phytocystatin Ayurvedic [2–4].

Generally, phytocystatins contain several conserved motifs such as QxVxG (the active site), a PW dipeptide in the region towards the C-terminal, and one or two residues of G towards the N-terminal. Another notable motif is the conserved [LVI]-[AGT]-[RKE]-[FY]-[AS]-[VI]-X-[EDQV]-[HYFQ]-N sequence which is found in the upstream of the active site. The sequence formed an *α*-helix structure but lacks disulphide bonds and glycosylation sites [6–9, 13, 14]. Phytocystatins identified so far have different sizes; the majority are small proteins that range from 12 kDa to 16 kDa and contain no disulphide bonds [6, 7, 10]. Several phytocystatins have slightly higher molecular masses, around 23 kDa, due to the C-terminal extension, containing the motif SNSL and they are reported to inhibit legumain [15]. In potato and tomato, multicystatins have been identified with multiple cystatin domains and have a molecular mass of 85 kDa [6, 7, 10].

Turmeric (*Curcuma *sp.), a medicinal plant from Zingiberaceae family, is one of such novel sources and up till now there are no PIs that are being characterized and reported. Turmeric is commonly used as traditional medicines and spices for culinary arts in country like India, Malaysia, and other Asian countries. Turmeric, as stated in Ayurvedic medicine, has the characteristic of antifungal and anti-inflammatory properties [16, 17]. Recent studies have also proven that turmeric does exhibit antitumour, antimicrobial, anti-HIV, nematocidal, and antioxidant properties [18–20]. Sookkongwaree et al. [21] had extracted secondary compounds from Zingiberaceae family and it was proven to exhibit antiviral and antiprotease properties. It is suggested that PIs could have possibly contributed to the antiviral and antiprotease properties of the secondary compounds.

In this study, we have identified and characterized a novel full-length sequence of cysteine protease inhibitor gene from* Curcuma longa* by using RACE-method (rapid amplification of cDNA ends). The phylogenetic relationship of the gene with other plant species and predictions of the putative functions of the identified gene was also conducted.

## 2. Results

### 2.1. Comparison of Total RNA Extraction with Different Methods

**Figure 1:**
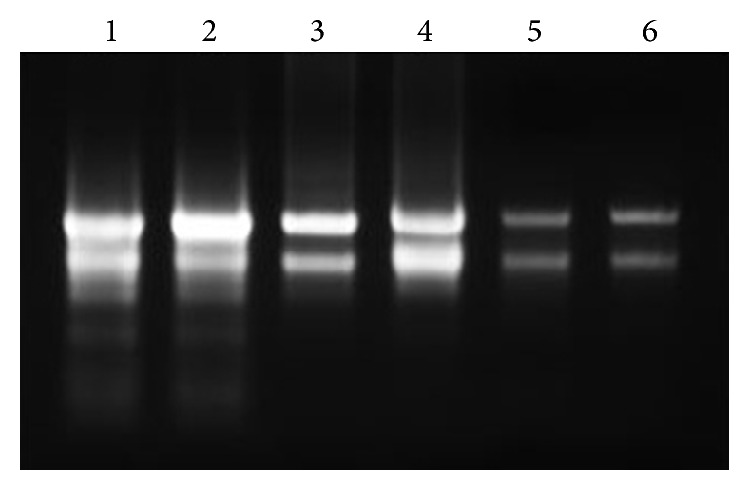
Total RNA integrity test on 1.0% agarose gel. Intact 28S and 18S total RNA bands can be observed on the agarose gel, indicating a good integrity of the total RNA after being extracted by different methods (lanes 1 and 2: modified CTAB method, lane 3, 4: RNAzol RT, and lane 5, 6: RNeasy Plant Mini kit).

Total RNA of turmeric (*Curcuma longa)* was successfully extracted from mature leaves of turmeric plant by three different methods: (i) modified CTAB (cetyltrimethyl ammonium bromide) method, (ii) RNAzol RT (Molecular Research Center Inc., USA), and (iii) RNeasy Plant Mini kit (Qiagen, USA). Intact bands of the 28S and 18S RNA can be clearly observed in the agarose gel for all the three methods (Figure 1). As for the quantitative test of the total RNA, with the raw tissue weight of 200 mg, our results showed that total RNA extraction by RNAzol RT produced the highest yield with concentration of ~800 ng/*μ*L while extraction by RNeasy Plant Mini kit and by a modified CTAB method yields ~300 ng/*μ*L of total RNA. Both RNAzol RT and RNeasy Plant Mini kit produced* A*_260/280 _ratio at the range of 1.70–1.80 while a modified CTAB method produced the ratio at the range of 2.00–2.20.  Table 1 summarised the comparison of the three extraction methods.

**Figure 2:**
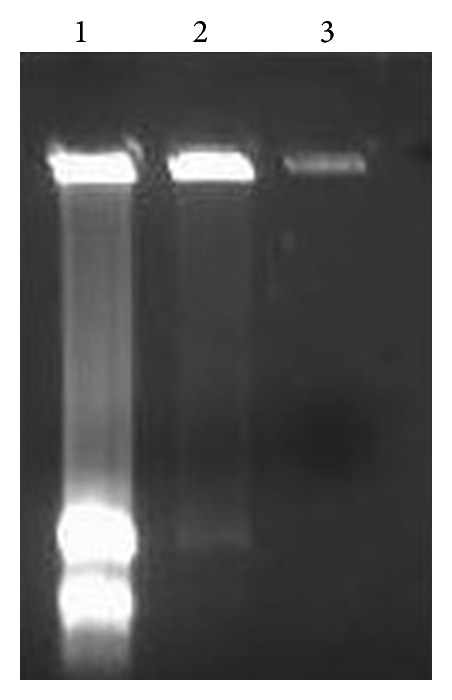
Total RNA extracted by using RNAzol RT on different tissues samples: (1) leaves, (2) flowers, and (3) rhizomes. The results showed that only the total RNA from the leaves samples was successfully extracted as compared to the other two samples.

Therefore, RNAzol RT was chosen as the preferred method to extract the total RNA from different parts of turmeric plant (leaf, flower, and rhizome) because of the highest extraction yield and faster time duration as compared to the other methods. However, based on the agarose gel electrophoresis result (Figure 2), it shows that RNAzol RT is only able to extract total RNA from leaves and is not suitable for flowers and rhizomes.

### 2.2. Cloning and Sequence Analysis of* Curcuma longa *Phytocystatin cDNA

**Figure 3:**
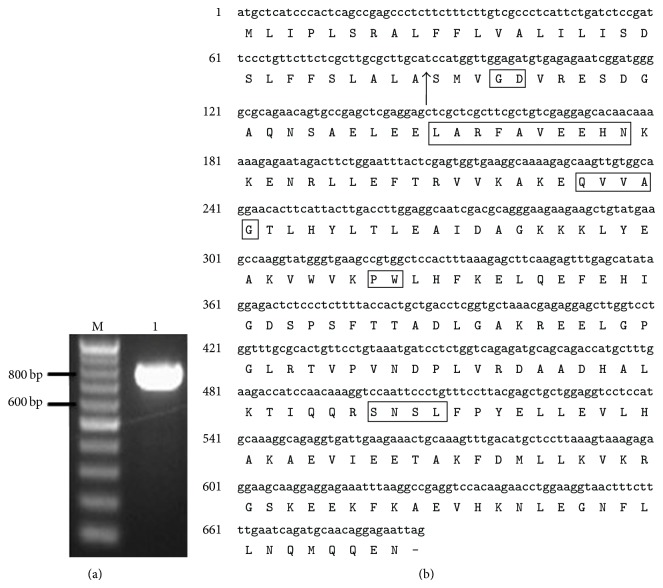
(a) cDNA of* CypC *with 870 bp long was amplified using primers covering from 5′ end to 3′ end designed from the information obtained in 5′/3′ RACE-method (lane M: 100 bp DNA ladder, lane 1:* CypCl*). (b) Nucleotide and deduced amino acid sequences of the identified full-length cDNA of* CypCl*. A signal peptide (from amino acid position 1–29th) was predicted by using SignalP3.0 program. The cleavage site is between amino acids 29 and 30 (as indicated with black arrow). Several motifs of phytocystatin were detected in the full-length sequence, such as (i) one G residues towards N-terminal, (ii) LARFAVEEHN, (iii) QVVAG, (iv) PW dipeptide, and (v) SNSL. The respective motifs had been boxed.

Degenerate polymerase chain reaction (PCR) was conducted to isolate the novel target gene, phytocystatin. A 495 bp cDNA fragment was successfully amplified from total cDNA reverse transcribed from the total RNA of leaves sample of* Curcuma longa*. 5′/3′ RACE (rapid amplification of cDNA ends) was conducted and the full-length cDNA designated* CypCl* was then deposited to the NCBI (National Center for Biotechnology Information) database with accession number KF545954.1. The cDNA (Figure 3(a)) is 870 bp long and contains a 687 bp long open reading frame (ORF) encoding 228 amino acids.

**Figure 4:**
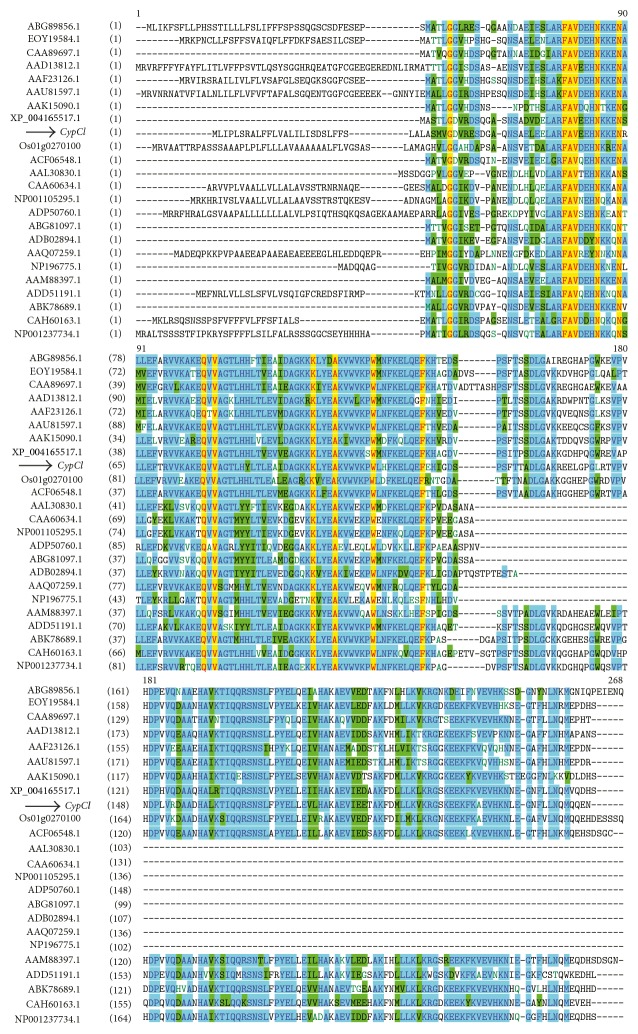
Multiple alignments of the deduced amino acids of the full-length sequence of* CypCl* (indicated with black arrow) with other phytocystatin sequences from other plants obtained from the GenBank database in the NCBI website. They were* Amaranthus hypochondiacus *(ABG89856.1),* Theobroma cacao *(EOY19584.1),* Ricinus communis* (CAA89697.1),* Ipomoea batatas *(AAD13812.1),* Solanum lycopersium *(AAF23126.1),* Petunia x hybrida *(AAU81597.1),* Sesamum indicum *(AAK15090.1),* Cucumis sativus *(XP_004165517.1),* Oryza sativa *japonica group (Os01g0270100),* Elaeis guineensis* (ACF06548.1),* Oryza sativa *(AAL30830.1),* Sorghum bicolour *(CAA60634.1),* Zea mays *(NP001105295.1),* Triticum aestivum x Secale cereal *(ADP50760.1),* Pelargonium x hortorum *(ABG81097.1),* Jatropha curcas *(ADB02894.1),* Ananas comosus *(AAQ07259.1),* Arabidopsis thaliana 1 *(NP19675.1),* Colocasia esculenta *(AAM88397.1),* Vitis cinerea var. helleri x Vitis riparia* (ADD51191.1),* Brassica rapa *(ABK78689.1),* Fragaria x ananassa *(CAH60163.1), and* Glycine max *(NP001237734.1).

**Figure 5:**
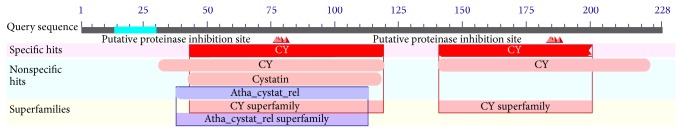
Graphical summary of BLAST results of the deduced amino acids of* CypCl* on conserved domains. It shows that several conserved cystatin superfamily domains are detected on the sequence suggesting a novel putative phytocystatin isolated from* Curcuma longa*.

Sequence analysis of the full-length* CypCl* gene (Figure 3(b)) showed that the deduced amino acid sequence of* CypCl* contained motifs that are generally found in phytocystatin. The detected motifs are (i) a single G residue and [LVI]-[AGT]-[RKE]-[FY]-[AS]-[VI]-X-[EDQV]-[HYFQ]-N sequence towards the N-terminal, (ii) QxVxG sequence, and (iii) PW dipeptide towards the C-terminal. Furthermore,* CypCl* is shown to contain C-terminal extension as multiple alignments of the deduced amino acid sequence of* CypCl* along with phytocystatins of other plant species show sequence homology (Figure 4). Additional evidence includes the detection of the motif SNSL at C-terminal proximity of the gene. The predicted pI of the putative protein is 5.72 and a calculated molecular mass is 25.8 kDa. A putative start codon is positioned at 22–24 and the stop codon is at the position of 724–726 (Figure 3(a)). When the deduced amino acid sequence was subjected to protein BLAST, using BLASTP (Basic Local Alignment Search Tool) [22], several conserved domains belonging to cystatin superfamily were detected on the sequence (Figure 5); BLASTP also showed that the sequence showed the highest similarity (74% max identity) to the predicted cysteine protease inhibitors from* Cucumis sativus *(accession no. XP_004165517.1) with query coverage of 88% and *E*value of 4*e* − 102.

### 2.3. Sequence Analysis and Phylogenetic Tree Construction

**Figure 6:**
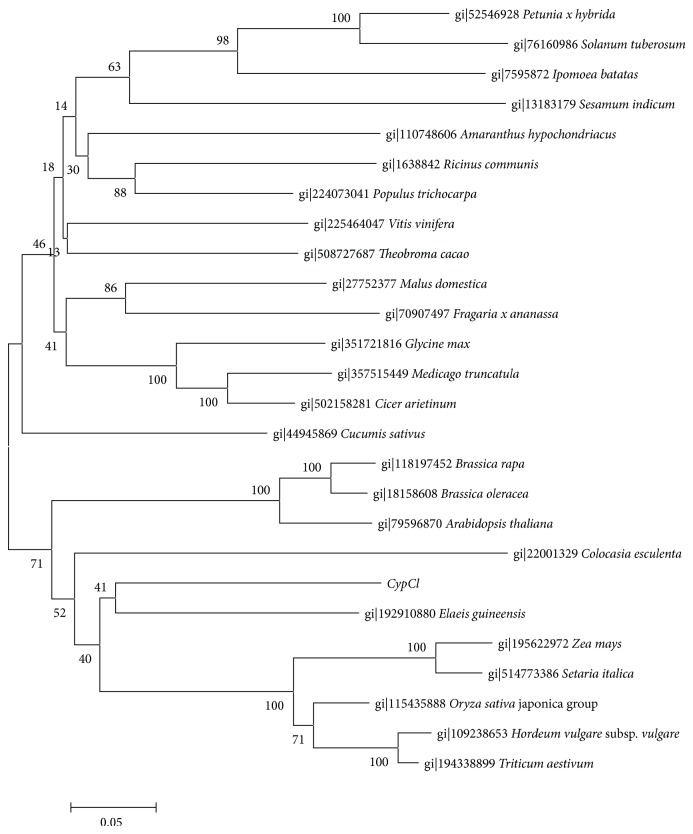
Phylogenetic tree generated with phytocystatins containing C-terminal extensions by using neighbour-joining method with 100 bootstrapping.* CypCl* is shown to be related to phytocystatin from* Elaeis guineensis *(African oil palm).

In order to study the evolution relationship and predicted function of the novel phytocystatin from turmeric, a phylogenetic tree was generated with deduced amino acid of* CypCl*. The unrooted phylogenetic tree (Figure 6) constructed with phytocystatin containing C-terminal extension by using neighbour-joining method showed that the novel* CypCl* from turmeric is related to phytocystatin from* Elaeis guineensis *(African oil palm).* CypCl* is grouped together with phytocystatins from other monocot plants forming one clade while the majority of eudicot plants formed another clade except for the Brassicaceae family.

With the presence of the motifs on the sequence, BLAST results, and the expected molecular mass (approximately 23 kDa), it is suggested that full-length cDNA,* CypCl*, is identified as phytocystatin and belongs to the group with the C-terminal extension. It is also suggested to be a novel phytocystatin and sequence is expected to exhibit the common functions of a phytocystatin with C-terminal extension.

## 3. Discussion

### 3.1. Total RNA Extraction with Different Methods

*Curcuma longa *plant is a medicinal plant that contains a high amount of secondary metabolites and phenolic compounds. In relation to that, three different extraction methods including one conventional method (CTAB method) and two commercially available kits (RNAzol RT and Qiagen RNeasy Plant Mini kits) were employed. The extraction result was compared to identify the most suitable method to extract total RNA from* Curcuma longa* for downstream applications and we had chosen RNAzol RT as the extraction method.

Based on the result in Figure 1 and Table 1, all of the methods successfully extracted total RNA from* Curcuma longa*'s leaves. However in terms of the speed of extraction, RNAzol RT and Qiagen RNeasy Plant Mini kits were fast and able to extract total RNA in less than an hour whereas CTAB method required 3 days to achieve the same result. Qiagen RNeasy Plant Mini kit utilises the silica-based membrane column to bind total RNA while RNAzol RT is a single-step total RNA extraction method which excludes the addition of chloroform to phase out the pure total RNA and the separation is done based on the interaction of phenol and guanidine with other cellular components [23]. CTAB method in comparison contained much more steps including a phenol : chloroform extraction, ethanol, and LiCl precipitation that lengthen the extraction period. While Qiagen RNeasy Plant Mini kit and RNAzol RT kit had similar speed in total RNA extraction, RNAzol produced a higher total RNA yield, about twice as much as yields of the other two methods.

However, the total RNA extracted by RNAzol RT and Qiagen RNeasy Plant Mini kits had a lower* A*_260/280 _ratio, from 1.70 to 2.00, compared to CTAB method with* A*_260/280 _ratio, from 2.00 to 2.20. A pure and good quality total RNA will have* A*_260/280 _ratio at 2.00 and the ratio below this value normally is caused by phenol or protein contamination. CTAB-based extraction method is a recommended method to extract total RNA from difficult samples including woody tissues and tissues rich in secondary metabolites. CTAB-based extraction buffer contained PVPP (polyvinyl polypyrrolidone) that helps in eliminating polyphenolics compounds from the homogenate by forming a complex through hydrogen bonding [24, 25]. The buffer also contained *β*-mercaptoethanol to prevent the production of quinones from phenolics, thus preserving RNA during the extraction [25]. However, the amount of secondary metabolites is higher in rhizome and flower samples than in leaves samples. This may explain why RNAzol RT method was unable to extract total RNA from the rhizome and flower samples as RNAzol RT lacked PVPP and *β*-mercaptoethanol which are crucial for total RNA extraction in samples with rich secondary metabolites [25]. Since only total RNA from leaves samples was needed for the isolation of* CypCl*, RNAzol RT method was chosen as the extraction method.

### 3.2. Analysis of Full-Length Phytocystatin mRNA in* Curcuma longa*

The full-length CypCl that was amplified from the cDNA of* Curcuma longa *was homology- analysed through BLAST against the existing phytocystatin genes in the GenBank and it was found to contain all the motifs that are common in phytocystatin family. The discovery of these motifs on the full-length sequence is crucial to identify and provide more information on the gene. The QVVAG motif and the PW dipeptide motif (Figure 3(b)) found on the full-length gene belong to the region of the protein that form hairpin loops, respectively, and are responsible for the inhibitory activity of cystatin [26]. By studying the structural model of oryzacystatin, a well- characterised phytocystatin from rice genome, both of the hairpin loops form a tripartite wedge with the conserved G residues (found on the N-terminal) which then slots into the active site cleft of the target enzyme and inhibits it. The role of the tripartite structural elements was confirmed when mutagenesis including amino acid substitution, partial deletions, random mutations, and mutant variants on these hairpin loops had showed reduction or termination of the inhibitory effect of cystatin towards papain [10]. The presence of these motifs on the amino acid sequence of the full-length* CypCl* suggested that the full length of gene is probably functionally active in papain inhibition.

In addition, the analysis of the full-length* CypCl* against other phytocystatins in the database also showed that it contained a conserved [LVI]-[AGT]-[RKE]-[FY]-[AS]-[VI]-X-[EDQV]-[HYFQ]-N motif, where the sequence in the cDNA is LARFAVEEHN, and the SNSL motif in C-terminal extension. The [LVI]-[AGT]-[RKE]-[FY]-[AS]-[VI]-X-[EDQV]-[HYFQ]-N conserved motif is found within the *α*-helix structure of phytocystatin but the function of this structure is still unknown [10, 26]. However, on the evolutionary point of view, this sequence could possibly shed some light on the evolutionary scheme of cystatins among the animals and plants. This sequence is generally found in phytocystatins but seldom to be found in animal cystatin which supports the theory of a common ancestor before the split between animals and plants [10]. While the SNSL sequence in C-terminal extension of the full-length* CypCl* is only found in some phytocystatins, phytocystatins with C-terminal extension containing this sequence are predicted to inhibit legumain activity. Pereira et al. [12] had proved that the SNSL site of the phytocystatins contained in the C-terminal extension is essential when phytocystatin with and without C-terminal extension was tested for legumain-inhibition activity; only those with C-terminal extension are able to inhibit legumain activity. Pereira et al. [12] also showed the importance of amino acid N (ASN, asparagine) in SNSL sequence as when it is replaced by a K (Lys, lysine) residue, the phytocystatin is unable to inhibit legumain. With the presences of the conserved sequences coding for the tripartite wedge responsible for papain inhibition and the C-terminal extension which is responsible for legumain inhibition, the novel* CypCl* could be expressed as a bifunctional protease inhibitor.

## 4. Materials and Methods

### 4.1. Plant Treatment and Sampling

Before sampling, C*urcuma longa* plant (around 6 months old) was treated with 500 *μ*L of methyl jasmonate diluted in 4500 *μ*L of ethanol (in 1 : 9 ratios) and placed inside a sealed growth chamber for 24 hours. The mixture was pipetted to a cotton swab and kept at the bottom of the growth chamber without having any direct contact with the plant [27–29]. After 24 hours, the leaves, flower, and rhizome samples were collected with sterilized blade, washed with distilled water to remove dirt, and grounded immediately in liquid nitrogen. About 200 mg of the ground samples was aliquoted in 2 mL microcentrifuge tubes, labeled, and proceeded with total RNA extraction.

### 4.2. Total RNA Extraction

Total RNA was extracted with a conventional method, CTAB method [30], and two commercially available kits, RNAzol RT (Molecular Research Center Inc., USA) and RNeasy Plant Mini kit (Qiagen, USA) according to the manufacturer's protocol, respectively. The quality and integrity of the total RNA extracted were analysed in agarose gel electrophoresis and were quantified with NanoDrop ND 1000 spectrophotometer (Thermo Scientific, USA).

### 4.3. Data Mining and Degenerate Primer Design

Transcriptomic data of phytocystatins from different plants species were gathered from the GenBank of NCBI website. The full mRNA sequences in nucleotide and amino acids were aligned using AlignX, Vector NTI Advance 11 (Invitrogen, USA). From the alignment, conserved regions were detected and a pair of degenerate primers was designed, with forward degenerate primer, Cys_F (5′-CTCGCTCGHTTCGCCGTCGAYGAG-3′), and reverse degenerate primer, Cys_R (5′-GTTCTTGTGHACYTCDACCTTGAA-3′).

### 4.4. cDNA Construction and the First Round Polymerase Chain Reaction (PCR)

cDNA was constructed with 1 *μ*g of extracted total RNA using iScript Reverse Transcription Supermix for RT-qPCR (BioRad, CA, USA) according to the manufacturer's protocol. The first round polymerase chain reaction (PCR) was carried out in a 20 *μ*L reaction using GoTaqFlexi DNA polymerase kit (Promega, USA) with the designed degenerate primers according to the manufacturer's instructions. PCR was performed on a thermocycler with the following parameters: an initial heating of PCR activation at 94°C for 3 minutes followed by 30 cycles of 94°C for 45 seconds, 42°C for 45 seconds, and 72°C at 45 seconds and a final extension at 72°C for 7 minutes. The 495 bp amplified PCR product was purified from gel using QIAquick PCR Purification kit (Qiagen, USA) and ligated into pGEM-T easy vector (Promega, USA). Then it was transformed into top 10* E. coli* chemically competent cells and cultured overnight on an ampicillin-containing Luria-Bertani (LB) agar, spread with 40 *μ*g/*μ*L X-gal and 40 *μ*L of 500 mM IPTG at 37°C. Blue/white colony screening was carried out and selected colonies were cultured overnight in ampicillin-containing LB broth. Plasmids were extracted using NucleoSpin Plasmid (Macherey-Nagel, Germany) according to the manufacturer's instructions and the plasmid was sent for sequencing at 1st base (Malaysia). The identity of the PCR product was determined by subjecting it to the Basic Local Alignment Search Tool (BLAST) [22] at NCBI website (http://blast.ncbi.nlm.nih.gov/).

### 4.5. Molecular Cloning of* CypCl* Gene and Sequence Analysis

Once the PCR product was proven to show similarity to other phytocystatins, gene-specific 5′ primer, Cyp5_R (5′-CTTGCTCTTTTGCCTTCACCACTC-3′) and 3′ primer, Cyp3_F (5′-TACGAGCTGCTGGAGGTCCTCCATGC-3′) were designed based on the sequence of the PCR product. To amplify 5′ cDNA end and 3′ cDNA end, RACE specific cDNA was generated using SMARTer RACE kit (Clontech, CA, USA) according to the manufacturer's protocol and 5′/3′ RACE was performed. From the obtained information on 5′ and 3′ cDNA end RACE, the full-length cDNA of* CypCl* covering the start codon until the stop codon was amplified using forward primer CypF_F (5′-GCTATCGAAGCGTGGCATCAT-3′) and reverse primer CypF_R (5′-GAGGTCACCCAAAGTCGTTACACA-3′). PCR conditions include initial activation at 94°C for 3 minutes followed by 35 cycles of 94°C for 45 seconds, 60°C for 45 seconds, and 72°C at 45 seconds and a final extension at 72°C for 10 minutes. The resulted PCR product was analysed under ultraviolet light on a 1.0% agarose gel (Vivantis, Malaysia) stained with ethidium bromide and a 100 bp DNA marker (Vivantis, Malaysia) was used. The band was purified, ligated in vector, and transformed into Top10* E. coli* competent cells as described in the previous step. The sequencing results were then subjected to BLAST and signal peptide analysis using SignalP 3.0 program.

### 4.6. Phylogenetic Tree Construction

Phytocystatins from other plants were gathered from NCBI website and aligned using MEGA 5.2 software [31]. The resulting alignment was trimmed and the alignment was subjected to phylogenetic tree construction by MEGA 5.2 software [31] using neighbour-joining method with 100 bootstrapping.

## Figures and Tables

**Table 1:** Efficiency of different methods of total RNA extraction from turmeric (*Curcuma *sp.) plant.

Method	*A*_260/280_ ratio	Yield concentration (ng/*μ*L)	Time duration
Modified CTAB Method	2.00–2.20	~300	Slow
RNAzol RT	1.80–2.00	800–1000	Fast
RNeasy Plant Mini kit	1.70–2.00	300–500	Fast
